# CD4+CD25+FoxP3+ Regulatory Tregs inhibit fibrocyte recruitment and fibrosis via suppression of FGF-9 production in the TGF-β1 exposed murine lung

**DOI:** 10.3389/fphar.2014.00080

**Published:** 2014-05-16

**Authors:** Xueyan Peng, Meagan W. Moore, Hong Peng, Huanxing Sun, Ye Gan, Robert J. Homer, Erica L. Herzog

**Affiliations:** ^1^Department of Internal Medicine, Section of Pulmonary, Critical Care, and Sleep Medicine, Yale School of MedicineNew Haven, CT, USA; ^2^Department of Respiratory Medicine, The Second Xiangya Hospital of Central-South UniversityChangsha, Hunan, China

**Keywords:** regulatory T cells, Fibrosis, TGF-β1, FGF-9, fibrocytes

## Abstract

Pulmonary fibrosis is a difficult to treat, often fatal disease whose pathogenesis involves dysregulated TGF-β1 signaling. CD4+CD25+FoxP3+ Regulatory T cells (“Tregs”) exert important effects on host tolerance and arise from naïve CD4+ lymphocytes in response to TGF-β1. However, the precise contribution of Tregs to experimentally induced murine lung fibrosis remains unclear. We sought to better understand the role of Tregs in this context. Using a model of fibrosis caused by lung specific, doxycycline inducible overexpression of the bioactive form of the human TGF-β1 gene we find that Tregs accumulate in the lung parenchyma within 5 days of transgene activation and that this enhancement persists to at least 14 days. Anti-CD25 Antibody mediated depletion of Tregs causes increased accumulation of soluble collagen and of intrapulmonary CD45+Col Iα1 fibrocytes. These effects are accompanied by enhanced local concentrations of the classical inflammatory mediators CD40L, TNF-α, and IL-1α, along with the neuroimmune molecule fibroblast growth factor 9 (FGF-9, also known as “glial activating factor”). FGF-9 expression localizes to parenchymal cells and alveolar macrophages in this model and antibody mediated neutralization of FGF-9 results in attenuated detection of intrapulmonary collagen and fibrocytes without affecting Treg quantities. These data indicate that CD4+CD25+FoxP3+ Tregs attenuate TGF-β1 induced lung fibrosis and fibrocyte accumulation in part via suppression of FGF-9.

## Introduction

Pulmonary fibrosis is a difficult to treat and life-threatening disease that is characterized by the dysregulated accumulation of extracellular matrix components in the parenchyma of the lung (Murray et al., 2012). This disorder is seen in the setting of autoimmune connective tissue disorders such as scleroderma (Homer and Herzog, 2010), non-inflammatory disorders such as gastrointestinal reflux disease (Lee et al., 2010), occupational exposures such as asbestos (Redlich et al., 2012), and cryptogenic forms such as idiopathic pulmonary fibrosis (IPF) (Raghu et al., 2011). Pulmonary fibrosis affects millions of people worldwide and the incidence is rising (Raghu et al., 2011). The development of pulmonary fibrosis is believed to involve a complex interplay between structural cell death responses and recruitment of inflammatory cells including collagen-producing fibrocytes. These events ultimately result in the activation of profibrotic growth factors such as TGF-β1 and culminate in myofibroblast transformation and ECM accumulation (Reilkoff et al., 2011). This paradigm renders TGF-β1-targeted therapies particularly attractive for the treatment of fibrotic lung disorders however given its central role in the regulation of cellular survival, proliferation, ECM maintenance, tumorigenesis, and immune regulation, direct inhibition of TGF-β1 might have undesired effects on tissue homeostasis (Massague, 2012). Thus, examination of factors that might be used to specifically regulate the pathogenic aspects of TGF-β1 signaling may facilitate better treatments for pulmonary fibrosis.

The contribution of CD4+ lymphocytes in general, and regulatory T cells in specific, to TGF-β1 -induced tissue remodeling remains unclear (Luzina et al., 2008). In terms of T cell responses, studies in lymphocyte deficient mice demonstrate that T cells are not required for the development of fibrosis and remodeling in the setting of experimentally induced pulmonary pathology (Helene et al., 1999). However, an emerging body of literature using sophisticated murine modeling indicates that discrete Thelper populations exert competing effects on development of fibrosis and recent studies in humans demonstrate that abnormalities in local and circulating T cell responses are associated with poor outcomes in IPF (Herazo-Maya et al., 2013; Reilkoff et al., 2013), thereby suggesting that the T cell contribution to lung fibrosis is likely more complex than previously thought. Current paradigms include a role for Th1 cells in the injury responses that are believed to initiate fibrosis and Th2 cells in the orchestration of subsequent remodeling and ECM (Wynn, 2004; Homer et al., 2011). Regulatory T cells which both respond to and produce TGF-β1 are thought to control peripheral host tolerance by modulation of immune responses. However, evaluation of these cells in the context of experimentally induced fibrosis has been challenging, with Tregs demonstrating fibrosis promoting (Liu et al., 2010; Lo Re et al., 2011) or fibrosis-limiting effects (Trujillo et al., 2010; Garibaldi et al., 2013) depending on the model and methods used. Furthermore, the specific contribution of Tregs to TGF-β1 induced lung fibrosis in specific, and the mechanisms through which these cells might orchestrate the tissue fibrotic response, remain important but unelucidated areas of lung biology.

Fibroblast Growth Factor-9, also called “FGF-9” or “glial activating factor” is a heparin binding, secreted growth factor that was originally described in glial cells (Miyamoto et al., 1993). Since its initial description in 1993 this protein has been detected in a wide variety of tissues where it exerts regulatory effects on such essential cellular processes embryonic development (Yin et al., 2011), cellular proliferation (Fakhry et al., 2005), and migration (Yu et al., 2012), tissue repair (Behr et al., 2010), and even tumor formation (Yin et al., 2013) and invasion (Teishima et al., 2012). FGF-9 is also known for its regulation of sex determination in the developing embryo (Bowles et al., 2010) and for its regulation of mesenchymal proliferation and epithelial branching in the developing mouse lung (Yin et al., 2011). In the normal adult mouse lung, FGF-9 protein is nearly undetectable (Yin et al., 2013). FGF-9 has also recently been linked to human lung fibrosis (Coffey et al., 2013) but its role in this disease has to date not been experimentally defined. In terms of immune regulation, FGF-9 has been only minimally studied but appears to be secreted by gamma delta T cells where it functions to regulate hair follicle regeneration (Gay et al., 2013). However, despite its involvement in multiple cellular processes that would be expected to regulate repair and remodeling, to date this protein has been little studied in the context of TGF-β1 induced immunopathogenetic responses in general.

To better understand these issues, we used transgenic mouse modeling and antibody mediated neutralization studies to define the contribution of Tregs to the development of experimentally induced lung fibrosis in the setting of lung specific, inducible transgenic TGF-β1 overexpression. Our results indicated that regulatory T cells exert suppressive effects on collagen accumulation and fibrocyte recruitment and that these effects are mediated, at least in part, via inhibition of FGF-9.

## Materials and methods

### Transgenic mice

All mouse experiments were approved by the Yale School of Medicine Institutional Animal Care and Use Committee. The CC10-tTS-rtTA-TGF-β1 transgenic mice used in this study have been described previously. These mice use the Clara cell 10-kDa protein (CC10) promoter to specifically express bioactive human TGF-β1 to the lung, and were backcrossed for >10 generations onto a C57BL/6 background (Lee et al., 2004)

### Doxycycline administration

CC10-tTS-rtTA-TGF-β1 (from hereon called “TGF-β1 mice”) transgene positive (Tg+) or their wild-type littermate controls (transgene negative, Tg−), age 8–10 weeks, were given 0.5 mg/ml doxycycline in their drinking water for up to 14 days (Gan et al., 2011).

### Neutralizing antibody administration

TGF-β1 Tg+ or TGF-β1 Tg− mice were injected intraperitoneally (i.p.) with neutralizing antibodies raised against CD25 (Li et al., 2008; Liu et al., 2010) or FGF-9 (Li et al., 2008). For CD25 neutralizing studies, 125 μg of anti-CD25 (Biolegend # 101906) or IgG2b isotype control (Biolegend #400622) was injected on days 4, 7, and 10 of doxycycline administration and mice were sacrificed on day 14. For FGF-9 blocking experiments, 250 μg of anti-FGF-9 (R&D #MAB273) or IgG2A isotype control (R&D #MAB003) were injected on days—1, 2, 5, 8, and 11 of doxycycline administration and mice were sacrificed on day 14.

### BAL and sacrifice

Euthanasia and bronchoalveolar lavage were performed as previously described (Gan et al., 2011).

### Flow cytometric analysis for fibrocytes

Following sacrifice of mice, the right upper (RUL) and right middle (RML) lobes were combined and digested for flow cytometry and total viable cells were quantified using Trypan blue staining as previously described (Gan et al., 2011). Flow cytometric analyses for Tregs and fibrocytes were performed according to our previously published methods. Antibodies against CD25, CD4, FOXP3, CD45, and appropriate isotype controls were obtained from BD Pharmingen. Antibody against Collagen-Iα was obtained from Rockland. Flow cytometry and cell sorting was performed using a BD FACSCalibur. Data were analyzed using Flow Jo v 7.5 software (TreeStar, Inc., Ashland, OR). For all analyses, isotype control staining was subtracted from true antibody staining to determine the percentage of positive cells. Fibrocyte detection was performed by first gating on CD45+ cells in the lung suspensions and then determining type Iα collagen (Col-Iα), content in these cells as we and others have described previously (Russell et al., 2012)The percentages of these cell present in each unit lung were multiplied by total viable cell count of mouse digested lung to determine the absolute number of CD45+Col-Iα+ cells per RUL and RML (RUL + RML).

### Sircol analysis

Right lower lobe (RLL) soluble collagen was measured using the Sircol Assay (Biocolor Ltd., UK) as we have previously described (Gan et al., 2011).

### Histologic analysis and immunohistochemistry

Whole left lungs were harvested from experimental mice for histological analysis. Formalin-fixed and paraffin-embedded sections were stained with hematoxylin and eosin to assess gross morphology or Masson's trichrome stains to visualize collagen deposition (Gan et al., 2011). FGF-9 immunohistochemistry was performed on unstained paraffin sections using anti-FGF-9 primary antibody followed by alkaline phosphatase-labeled secondary. Staining was visualized via application of the red chromogenic substrate (Dako #K60404).

### Multianalyte ELISA

Cytokine measurements were performed on BAL fluid using Luminex technology as previously described (Gaschler et al., 2009). Human TGF-β1 concentration was measured by ELISA (R&D # DB100B).

### Statistical analysis

Normally distributed data are expressed as mean ± s.e.m. and assessed for significance by Student's *t*-test or ANOVA as appropriate with Bonferroni post-test. Data that were not normally distributed were assessed for significance using the Mann-Whitney *U*-test where appropriate.

## Results

### CD4+CD25+FoxP3+ tregs accumulate in the TGF-β1-exposed murine lung

**Figure 1:**
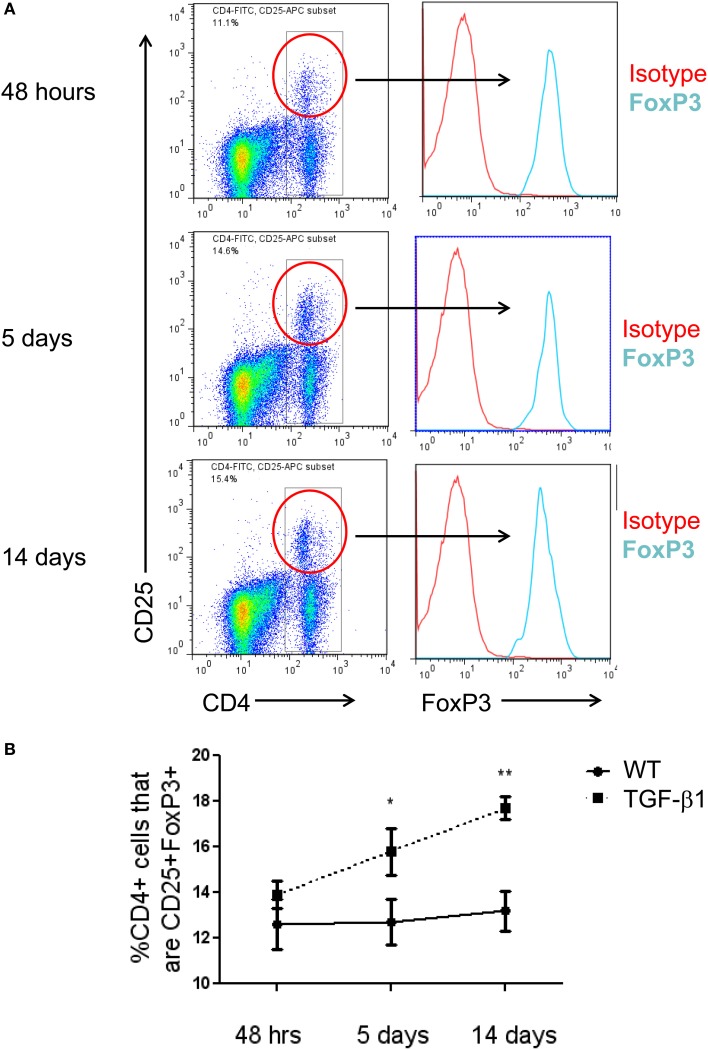
**CD4+CD25+FoxP3+ Tregs accumulate in the TGF-β1-exposed murine lung. (A)** Flow cytometric detection of Tregs in the TGF-β1 Tg+ lung. TGF-β1 Tg+ and Tg− (not shown) mice were administered doxycycline in their drinking water for 2, 5, or 14 days, sacrificed, and flow cytometry was performed on lung digests. Live cells were analyzed and percent of cells triple positive for CD4, CD25, and FoxP3 was determined as shown. One representative mouse sample from each group of TGF-β1 Tg+ mice is depicted. **(B)** Comparison of Treg percentages in TGF-β1 Tg+ and Tg− lungs at early and late time points during transgene activation. ^*^p < 0.05 compared to the 48 h time point. ^**^p < 0.01 compared to the 48 h time point. *n* > 4 mice/group, performed in triplicate.

In order to determine whether Tregs accumulate in the lungs of mice subject to TGFβ1-induced lung fibrosis, TGF-β1 transgene positive (Tg+) and transgene negative (Tg−, i.e., wild type) mice were given doxycycline in their drinking water, sacrificed at early (48 h), intermediate (5 days), and late timepoints (14 days) and Tregs were quantified using standard FACS-based assessment of CD4+CD25+FoxP3+ cells on lung digests (Figure 1A). Using this approach we found that the percentage of CD4+ cells meeting flow cytometric criteria for Tregs were unchanged at the early timepoint but that compared to Tg− mice, the lungs of Tg+ mice exposed to 5 days of doxycycline contained a 24.3% increase in the percentage of CD4+ cells that were Tregs (*p* = 0.0252, Figure 1B) that was further increased to 34.1% at 14 days (*p* = 0.0021, Figure 1B). There was no difference in TGF-β1 induced Treg percentages between day 5 and 14 (Figure 1B). These data indicate that Tregs accumulate in the TGF-β1 exposed murine lung during the period of fibrogenesis that is dominated by inflammatory cell recruitment and fibroblast activation, suggesting that the potential role of Tregs in fibrosis might relate to one or both of these processes.

### Antibody mediated depletion of tregs worsens TGF-β1 induced lung fibrosis

**Figure 2:**
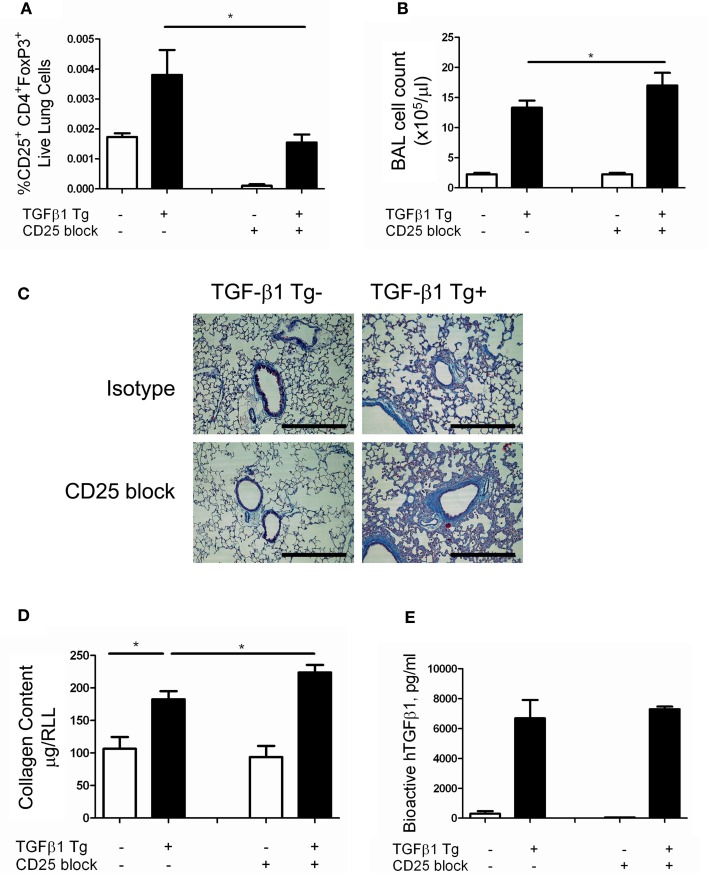
**Depletion of Tregs exacerbates TGF-β1 induced lung fibrosis**. TGF-β1 Tg+ and Tg− mice were administered doxycycline for 14 days during which they were injected i.p. with anti-CD25 antibody on days 4, 7, and 10. All mice were sacrificed on day 14, BAL was performed, and lungs were processed for flow cytometry, Sircol assay, and histology as described in the Methods. **(A)** Percentage of live lung cells that are CD25+/CD4+/FoxP3+ as determined by flow cytometry performed on combined right upper and middle lobes. **(B)** BAL leukocyte cell concentrations (absolute # per μl). **(C)** Trichrome staining of paraffin embedded lung sections from TGF-β1 Tg+ and Tg− mice treated with anti-CD25 antibody or isotype control. Scale bar = 250 microns. **(D)** Lung collagen content measured by Sircol assay performed on the right lower lobe (RLL). **(E)** Human (transgenic) bioactive TGF-β1 concentrations in mouse BAL fluid as determined by ELISA. ^*^p < 0.05. *n* > 4 mice/group, performed in triplicate.

Tregs have been reported to have competing effects in several models of lung fibrosis caused by inhalational bleomycin, LPS administration, and chronic silica exposure. The data described above demonstrate a modest increase in Treg accumulate in the TGF-β1 exposed mouse lung. In order to determine if this enhancement of Tregs is biologically relevant in our model, TGF-β1 Tg− and Tg+ mice were exposed to doxycycline and treated with a well characterized anti-CD25 antibody that has been previously used for these types of studies (Liu et al., 2010). Because an increase in Tregs was first detected at 5 days of transgene activation we initiated dosing on day 4 and repeated antibody administration every 72 h until the mice had received 14 days of doxycycline at which point the mice were sacrificed. Flow cytometry confirmed depletion of most Tregs in this model (Figure 2A). Analysis of samples obtained from these animals demonstrate that CD25 neutralization increases total lung inflammatory cell content based on BAL cell counts (Figure 2B). Histologic evaluation of fibrosis performed using Masson's Trichrome stains found enhanced collagen accumulation seen both around the airways and in the alveoli of Treg depleted TGF-β1 mice in comparison to isotype control TGF-β1 Tg+ mice (Figure 2C). Lung collagen content assessed by Sircol assay was increased by 21.2% in the lungs of Treg-depleted TGF-β1 Tg+ mice (*p* = 0.020, Figure 2D). Importantly, concentrations of bioactive hTGF-β1 did not differ between these groups (Figure 2E), indicating that the moderate increase in fibrosis seen in this setting is not related to an increase in the transgenic fibrotic stimulus. These data indicate that CD25-mediated Treg depletion leads to increased lung inflammation and fibrosis, thereby suggesting that immunoregulatory factors might be mediating these effects.

### Antibody mediated depletion of tregs increases intrapulmonary fibrocytes

**Figure 3:**
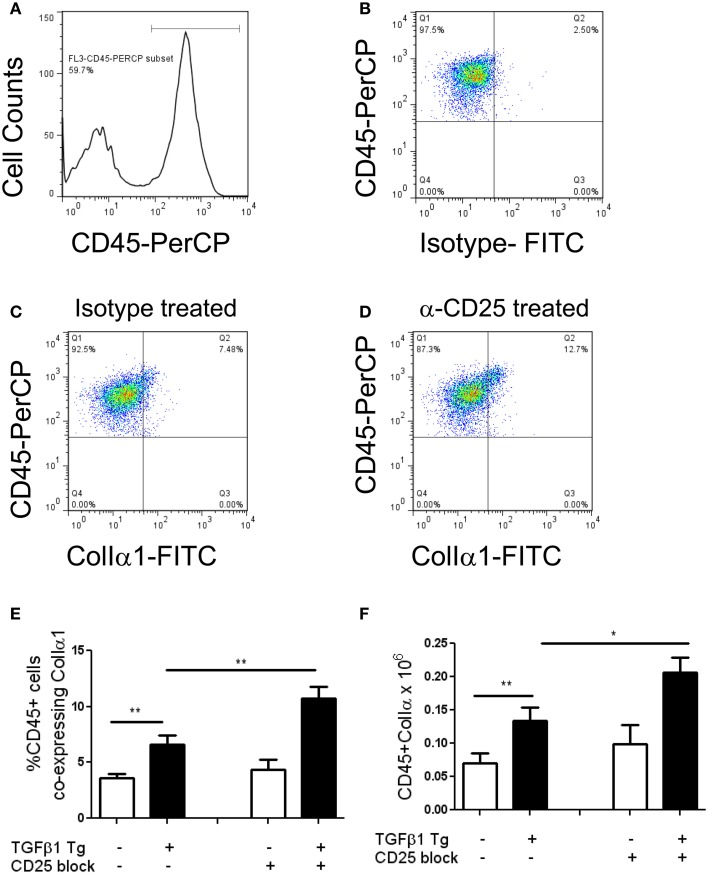
**Depletion of Tregs increases intrapulmonary fibrocytes in the TGF-β1 exposed murine lung**. Fibrocyte detection by flow cytometry of digested lungs from TGF-β1 Tg+ mice given doxycycline for 14 days and treated with isotype and anti-CD25 antibody. Panel **(A)** demonstrates the CD45+ population that was selected for analysis and Panel **(B)** presents FITC-detected intracellular isotype control (X axis) vs. CD45-PerCP (Y axis) that was used to determine the negative gate. **(C,D)** CD45+ ColIα1+ dot plots in lung digests obtained from doxycycline treated TGF-β1 Tg+ mice treated with **(C)** isotype control or **(D)** CD25 neutralizing antibody. Percentage **(E)** and concentration **(F)** of CD45+ ColIα1+ cells in lung digests of TGF-β1 Tg+ and Tg− mice treated with anti-CD25 antibody or isotype control. ^*^p < 0.05, ^**^p < 0.01. *n* > 4 mice/group, performed in triplicate.

Regulatory T cells are reported to regulate the appearance of fibrocytes (Garibaldi et al., 2013) in the setting of fibroproliferative ARDS. Given the association of fibrocytes and pulmonary fibrosis (Reilkoff et al., 2011), we thought it possible that the increased collagen accumulation seen in the setting of Treg depletion might be related to enhanced recruitment of fibrocytes. In order to test this hypothesis, flow cytometry was performed on lung digests prepared from TGF-β1 Tg+ and Tg− mice that did and did not receive CD25 mediated Treg depletion and enumeration of fibrocytes was performed following 14 days of transgene activation. Here we found that the increase in Tg+ lung fibrosis seen in the setting of CD25 depletion is accompanied by a 1.61-fold increase in the percentage of CD45+ cells that are Col Iα1+ fibrocytes (*p* = 0.04 Figures 3A–E) and a 1.56-fold increase in the absolute quantities of these cells (*p* = 0.0445, Figure 3F). These data indicate that removal of Tregs creates conditions in the TGF-β1 exposed murine lung that favor the accumulation of fibrocytes.

### Antibody mediated depletion of tregs augments soluble mediators of inflammation and FGF-9 expression

**Figure 4:**
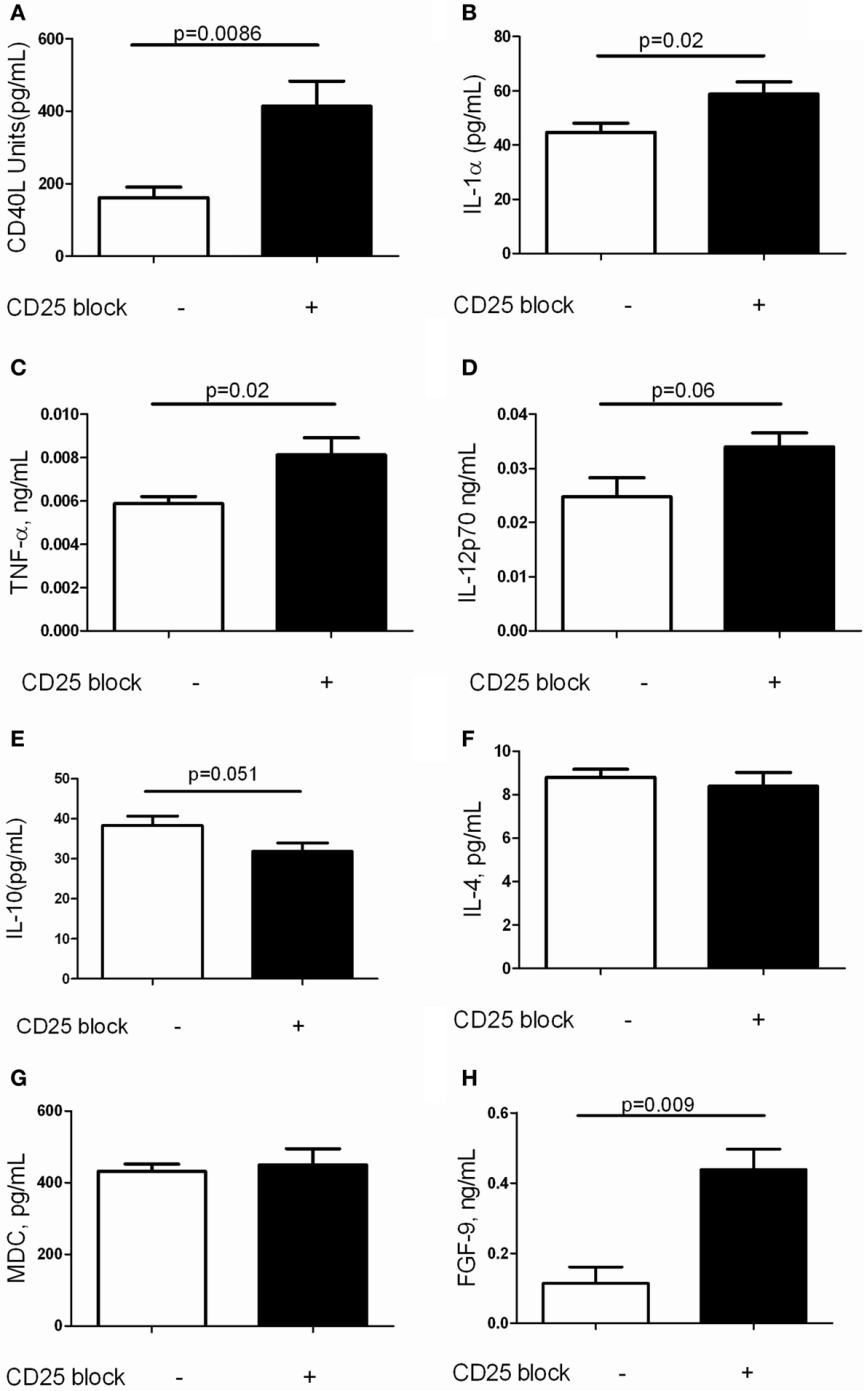
**Depletion of Tregs amplifies expression of inflammatory markers and FGF9 in the TGF-β1 exposed mouse lung**. Soluble mediators in the BAL fluid of TGF-β1 Tg+ mice given doxycycline for 14 days and treated with isotype or anti-CD25 neutralizing antibody were quantified by Luminex technology as described. **(A)** CD40L. **(B)** IL-1alpha. **(C)** TNF-alpha. **(D)** IL-12p70. **(E)** IL-10. **(F)** IL-4. **(G)** MDC. **(H)** FGF-9. Full results are presented in Table 1. *P*-values are indicated for each marker. *n* > 4 mice/group.

It is increasingly recognized that a complex inflammatory milieu exists in the fibrotic lung (Homer et al., 2011). Thus, assessments of BAL cell counts and perhaps even flow cytometric assessment of lung inflammation may not provide adequate insight into the factors associated with TGF-β1 induced fibrosis. In order to assess the concentrations of the soluble mediators that are influenced by Treg depletion in this model, we performed Luminex based quantification of BAL proteins in TGF-β1 Tg+ mice that did and did not receive CD25 blocking antibody. This well validated platform has been used successfully for unbiased biomarker discovery in humans (Richards et al., 2012) and for detection of novel therapeutic pathways in mice (Gaschler et al., 2009). Here we were not surprised to find that removal of Tregs substantially increased concentrations of the TNF superfamily member and costimulatory molecule CD40 ligand (Figure 4A) as well as the proinflammatory mediators IL-1α (Figure 4B) and TNF-α (Figure 4C). There was a trend toward increased IL-12p70 (Figure 4D) while the concentrations of IL-10 (Figure 4E), IL-4 (Figure 4F) and the alternatively activated macrophage marker MDC (Figure 4G) were unchanged when Tregs were depleted. We did note an unexpected increase in FGF-9 (Figure 4H), a neuroimmune molecule with manifold effects on embryonic development that may be implicated in human lung fibrosis (Coffey et al., 2013). The full results of these studies are presented in Table 1. These data indicate that Treg depletion increases concentrations of T cell activation markers and classical inflammatory mediators without affecting Th2 cytokines in the TGF-β1 exposed murine lung. In addition, these effects are accompanied by increased expression of FGF-9.

**Table 1:** ***P*-Values of BAL multiplex comparing isotype vs. anti-CD25 treated Tg+ mice**.

**Analyte**	***P*-value**
Apolipoprotein A1	0.547
CD40	0.327
CRP	0.807
Endothelin-1	0.681
Eotaxin	0.932
Epidermal growth factor	0.989
Factor VII	0.98
Fibrinogen	0.199
Basic FGF	0.566
GST-alpha	0.075
GM-CSF	0.994
KC/GRO	0.091
Haptoglobin	0.037
IgA	0.432
Interferon gamma	0.324
IP-10	0.416
IL-1β	0.782
IL-11	0.15
IL-12p70	0.268
IL-17A	0.341
IL-18	0.964
IL-2	0.258
IL-3	Undetectable
IL-5	0.384
IL-6	0.417
IL-7	0.423
LIF	0.697
Lymphotactin	0.7633
M-CSF-1	0.295
MIP-1α	0.912
MIP-Iβ	0.465
MIP-1g	0.164
MIP-2	0.546
MIP-3β	0.936
MDC	0.729
MMP-9	0.905
MCP-1	0.619
MCP-3	0.096
MCP-5	0.174
MPO	0.329
Myoglobin	0.064
OSM	0.238
SAP	0.968
SGOT	Undetectable
SCF	0.4066
RANTES	0.350
TPO	0.387
TF	0.139
TIMP-1 mouse	0.123
VCAM-1	0.964
VEGF-A	0.785
vWF	0.258

Analytes with significant changes are reported in the text.

### FGF-9 expressing cells are increased in the TGF-β1 exposed murine lung

**Figure 5:**
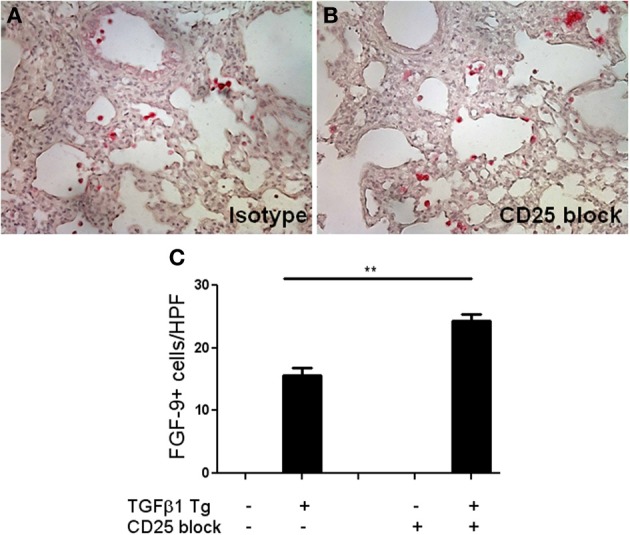
**Treg depletion results in increased accumulation of FGF-9 expressing cells in the fibrotic mouse lung. (A,B)** Immunohistochemisty for FGF-9 (red) on TGF-β1 Tg+ mice given doxycycline for 14 days and treated with isotype control or anti-CD25 antibody. Tissue sections were counterstained with hematoxylin. **(C)** Composite data represented as number of FGF-9+ cells per high power field. *n* > 4 mice/group. ^**^p < 0.01.

FGF-9, or glial activating factor, is a neuronal protein that regulates gonadal differentiation in the developing embryo (Dinapoli et al., 2006). This molecule is also a potent mitogen with significant stimulatory effects on a wide variety of biological processes including lung development *in utero* (Colvin et al., 2001) and tumorigenesis (Yin et al., 2013). While increased detection of FGF-9 has been reported in lungs of patients with IPF (Coffey et al., 2013) a role for FGF-9 in the regulation of experimentally induced lung fibrosis has yet to be described. In order to determine whether the increased FGF-9 seen in the setting of Treg depletion might be pathogenic, we used immunohistochemistry to determine the site of expression of FGF-9 in the setting of TGF-β1 overexpression (Figures 5A–C). Here we found that, consistent with prior reports, FGF-9 expression was undetectable in the naïve Tg—adult mouse lung (Figure 5C). In contrast, in the setting of transgenic TGF-β1 overexpression, immunodetection of FGF-9 was markedly increased in cells bearing the morphology of alveolar macrophages and epithelial cells (Figures 5A,B). Moreover, we detected a 1.75-fold fold increase in the quantity of FGF-9+ cells in the lungs of TGF-β1 Tg+ mice treated with anti-CD25 antibody relative to those administered isotype control (*p* = 0.007, Figure 5C), indicating that Treg depletion increases the presence of FGF-9+ cells in this experimental system.

### Antibody mediated neutralization of FGF-9 ameliorates TGF-β1 induced pulmonary fibrosis

**Figure 6:**
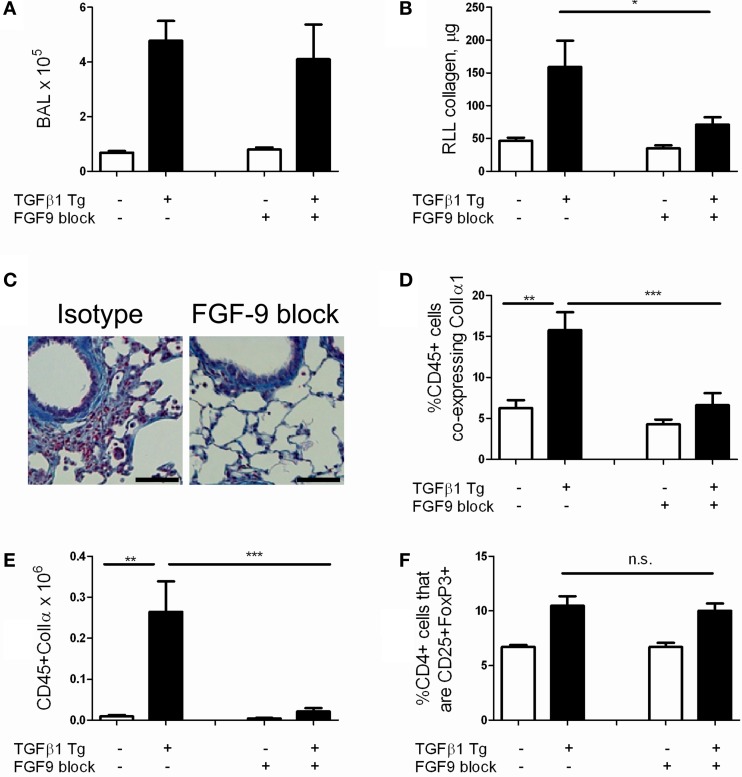
**FGF-9 blockade reduces lung fibrocyte accumulation and fibrosis in TGF-β1 transgenic mice**. TGF-β1 Tg+ and Tg− mice were administered 14 days of doxycycline and injected i.p. with either FGF-9 neutralizing antibody or isotype control on days—1, 2, 5, 8, and 11. **(A)** BAL leukocyte concentration (absolute # per μl). **(B)** Lung collagen content as measured by Sircol assay performed on the right lower lobe (RLL). **(C)** Trichrome staining of paraffin embedded lung sections from TGF-β1 Tg+ mice treated with isotype control (left) or anti-FGF-9 antibody (right). Scale bar = 100 microns **(D,E)** Percentages **(D)** and absolute number **(E)** of fibrocytes (CD45+ColIα1+) in lung digests as determined by flow cytometry. **(F)** Quantity of Tregs (expressed as percentage of CD4 cells that are CD25+FoxP3+) in lung digests as determined by flow cytometry. *n* = 5 mice/group. ^*^p < 0.05, ^**^p < 0.01, ^***^p < 0.001.

We next sought to assess whether FGF-9 promotes TGF-β1 induced lung fibrosis in our model. Because FGF-9 null mice are embryonic lethal (Colvin et al., 2001) we employed antibody mediated neutralization of FGF-9 using a well validated blocking antibody approach (Li et al., 2008). Here, TGF-β1 Tg− and Tg+ mice were randomized to receive intraperitoneal injections of FGF-9 neutralizing antibody or isotype control every 72 h from the night prior to doxycycline initiation until 14 days at which point they were sacrificed. Using this approach we found no reduction in BAL cell counts (Figure 6A) and a 55.3% reduction in lung collagen content (*p* = 0.027, Figure 6B). Examination of lung histology confirmed these findings (Figure 6C). These data indicate that FGF-9 neutralization reduces collagen accumulation and fibrosis in the TGF-β1 exposed murine lung without affecting inflammation.

### Antibody mediated neutralization of FGF-9 reduces detection of fibrocytes in the TGF-β1 exposed murine lung

Last, in order to determine whether FGF-9's effects on fibrosis extend to an immunomodulatory role as well, we quantified fibrocytes in the lungs of TGF-β1 Tg− and Tg+ mice that were subject to FGF-9 neutralization. Here, compared to sham-treated mice, we found a 57.5% reduction in percentages of CD45+ cells that co-express Col Iα1+ (*p* = 0.009, Figure 6D) and more than twelve-fold reduction in total quantities of these cells (Figure 6E, *p* = 0.008) in the lungs of TGF-β1 Tg+ mice that were treated with FGF-9 blockade. Importantly, percentages of Tregs were not influenced by this intervention (Figure 6F), indicating that FGF-9's effects on fibrosis and fibrocytes are enacted downstream of Tregs.

## Discussion

These data lend new insight into the mechanism(s) through which Tregs might regulate experimentally induced pulmonary fibrosis. Specifically, they demonstrate that in the setting of inducible overexpression of TGF-β1, enhanced quantities of Tregs are detected within 5 days of transgene activation and that this increase is sustained until at least 14 days. Antibody mediated depletion of Tregs leads to accumulation of collagen and increased detection of intrapulmonary fibrocytes. These findings are accompanied by a local increase in mediators associated with T cell activation and Th1 inflammation, as well as increased concentrations of FGF-9 in the TGF-β1 exposed murine lung. Antibody mediated FGF-9 neutralization reduces TGF-β1 induced fibrosis and accumulation of fibrocytes. These data present evidence that Tregs attenuate TGF-β1 induced lung fibrosis and fibrocyte accumulation in part via suppression of FGF-9. While FGF-9 has been shown to be expressed in fibrotic human lung and human fibroblasts in response to TGF-β1 (Coffey et al., 2013) the studies presented here provide a putative mechanistic role for this molecule in pulmonary fibrosis via the accumulation of fibrocytes within the TGF-β1 exposed lung.

The study of Tregs in experimentally induced lung fibrosis has yielded conflicting and at times contradictory results. For example, studies involving cytokine manipulation or Treg transfers in bleomycin fibrosis (Trujillo et al., 2010) or LPS induced acute lung injury (Garibaldi et al., 2013) models demonstrate a protective role for these cells. In contrast, several studies using the silicosis model indicate that Tregs possess profibrotic properties (Liu et al., 2010; Lo Re et al., 2011). The disparity of these findings is not surprising when one considers the experimental variance in models used and methods of study. In our work, we find that antibody mediated Treg removal worsens fibrosis and that these effects are accompanied by an increase in classical inflammatory mediators such as TNF-α and IL1-α. The source of these mediators is not entirely clear but because similar studies performed in LPS-treated Rag null mice demonstrate similar findings (Aggarwal et al., 2010), it is likely that this effect is at least partially T cell independent and relates to increased production by macrophages and structural cells such as the airway and alveolar epithelia. Because we also find an increase in classical inflammatory mediators in the setting of Treg depletion, we believe that Tregs function at the tissue level to cease damage and restore homeostasis via modulation of tissue inflammation and/or via direct engagement of local cytoprotective responses (D'Alessio et al., 2009). This concept is important because it suggests that rather than simply amplifying existing cell death responses, an absence of functional Tregs might permit or perhaps even induce tissue injury in the setting of profibrotic stimuli. Whether this conclusion will hold true when studied in other modeling systems and, more importantly, in the context of human disease, remains to be seen.

Our study indicates that Treg removal causes enhanced intrapulmonary accumulation of CD45+ ColIα1+ fibrocytes. Fibrocytes are a leukocyte derived mesenchymal cell population that are implicated in the immunologic events leading to the development of tissue fibrosis (Reilkoff et al., 2011). The data presented herein suggest that the enhanced local levels of FGF-9 caused by Treg depletion create a situation favoring the differentiation, recruitment, and/or retention of fibrocytes. In our system the Tregs do not themselves produce FGF-9, but rather modulate its production by structural cells and/or alveolar macrophages. To our knowledge, these are the first data to propose a role for either FGF-9 or alveolar macrophages in the regulation of fibrocyte accumulation. It is also possible that the increased levels of CD40L seen in our model stimulate a proinflammatory fibrocyte phenotype characterized by secretion of TNF-α as has been recently described in the setting of Grave's ophthalmopathy (Gillespie et al., 2012), though this hypothesis will require more evaluation. Further work will be required to dissect the precise relationship between Tregs, macrophages, fibrocytes, and inflammation in the context of fibroproliferative lung disease.

FGF-9 has been previously shown to regulate TGF-β1 induced cell proliferation in the developing palate (Iwata et al., 2012) and is induced in human lung fibroblasts in response to TGF-β1 stimulation (Coffey et al., 2013). Given its well documented stimulatory effects on organ development, cell proliferation, and angiogenesis it is not surprising that FGF-9 would be involved in tissue injury and pathologic remodeling. In the developing lung, FGF-9 engages FGFR2 and FGFR3 to regulate epithelial branching and mesenchymal proliferation via differentially regulated Wnt signaling (Yin et al., 2011). The nature of FGF-9 signaling in the setting of experimentally induced fibrosis remains uneludicated but may involve regulation of cell death responses, immunomodulation, or regulation of fibroblast proliferation. FGF-9 has been reported to be expressed in hyperplastic epithelial cells, myofibroblasts, and perivascular smooth muscle cells in the lungs of patients with IPF. To date, expression on inflammatory cells in the setting of tissue fibrosis has not been reported. Indeed, our detection of FGF-9+ alveolar macrophages in the context of an inflammatory milieu is consistent with previous findings that FGF-9 expression is enhanced in settings characterized by increased expression of IL-1α (Ueng et al., 2005). However, several questions remain. For example, we have not determined the receptor(s) or mechanism(s) through which FGF-9 regulates TGF-β1 induced fibrosis and fibrocyte accumulation though it is likely that FGFR2 and/or FGFR3 are involved. We have not defined the macrophage subpopulation(s) that produce FGF-9 in this model and we also have not ruled out expression by other cell types, such as gamma delta T cells, at different time points. Nevertheless, our data indicate that FGF-9 joins other neuroimmune molecules such as Semaphorin 7a (Reilkoff et al., 2013) and Plexin C1 (Gan et al., 2011) as important regulators of TGF-β1 associated fibrosis and/or fibrocyte accumulation.

In conclusion, these studies frame Tregs as important suppressors of TGF-β1 induced lung pathology and reveal FGF-9 as a downstream mediator of Treg activity. Further studies in this area might facilitate better understanding of tissue repair and remodeling.

## Author contributions

All authors participated in the conception or design of the work, acquisition, analysis, or interpretation of data, and final approval of the version to be published. Erica L. Herzog and Meagan W. Moore were responsible for the drafting and revising of the manuscript for important intellectual content. All authors agree to be accountable for all aspects of the work and to ensure that questions related to the accuracy or integrity of any part of the work are appropriately investigated and resolved.

## Funding sources

NIH HLR01-109033 (Erica L. Herzog)

### Conflict of interest statement

The authors declare that the research was conducted in the absence of any commercial or financial relationships that could be construed as a potential conflict of interest.
